# Evaluating oral epithelial dysplasia classification system by near-infrared Raman spectroscopy

**DOI:** 10.18632/oncotarget.19343

**Published:** 2017-07-18

**Authors:** Bo Li, Zhi-Yu Gu, Kai-Xiao Yan, Zhi-Ning Wen, Zhi-He Zhao, Long-Jiang Li, Yi Li

**Affiliations:** ^1^ State Key Laboratory of Oral Disease, West China Hospital of Stomatology, Sichuan University, Chengdu 610041, China; ^2^ Department of Head and Neck Oncology, West China Hospital of Stomatology, Sichuan University, Chengdu 610041, China; ^3^ College of Chemistry, Sichuan University, Chengdu 610064, China

**Keywords:** oral epithelial dysplasia, near-infrared Raman spectroscopy, support vector machine, classification system

## Abstract

Until now, the classification system of oral epithelial dysplasia is still based on the architectural and cytological changes, which relies on the observation of pathologists and is relatively subjective. The purpose of present research was to discriminate the oral dysplasia by the near-infrared Raman spectroscope, in order to evaluate the classification system. We collected Raman spectra of normal mucosa, oral squamous cell carcinoma (OSCC) and dysplasia by near-infrared Raman spectroscope. The biochemical variations between different stages were analyzed by the characteristic peaks in the subtracted mean spectra. Gaussian radial basis function support vector machines (SVM) were used to establish the diagnostic models. At the same time, principal component analysis (PCA) and linear discriminant analysis (LDA) were used to verify the results of SVM. Raman spectral differences were observed in the range between 730~1913 cm^-1^. Compared with normal mucosa, high contents of protein and DNA in oral dysplasia and OSCC were observed. There were no significant or gradual variation of Raman peaks among different dysplastic grades. The accuracies of comparison between mild, moderate, severe dysplasia with OSCC were 100%, 44.44%, 71.15%, which elucidated the low modeling ability of support vector machines, especially for the moderate dysplasia. The analysis by PCA-LDA could not discriminate the stages, either. Combined with support vector machines, near-infrared Raman spectroscopy could detect the biochemical variations in oral normal, OSCC and dysplastic tissues, but could not establish diagnostic model accurately. The classification system needs further improvements.

## INTRODUCTION

Dysplastic features of the oral epithelium are characterized by cellular atypia and loss of normal maturation and stratification [1]. The presence of dysplastic areas in the oral mucosa is believed to be associated with the progression to cancer. The severer the dysplasia is, the greater the likelihood of progression to malignancy in an individual lesion is. However, non-dysplastic lesions may also show malignant development [2, 3]. Therefore, presence and severity of dysplasia cannot be used as a reliable guide for the treatment of individual cases. Nevertheless, the crude relationship between grading dysplasia and risk of progression to malignancy makes dysplasia grading necessary [4].

Grading of dysplasia, including head and neck lesions, continues to be a hotly debated subject. It is subjective and it lacks intra- and inter-observer reproducibility due to the insufficiency of validated morphological criteria and the biological nature of dysplasia [3, 5]. With the increasing proliferative ability of the dysplastic cells, the contents and types of DNA and proteins will change, which could induce spectral differences of cells and tissues [6]. So if there existed any significant biochemical variations among the different dysplastic grades, the lesions could be discriminated by the Raman spectroscope.

In the past decades, Raman spectroscopy, which makes use of an inelastic light scattering process to capture ‘fingerprints’ of specific molecular structures and conformations of a given tissue or disease state, have been comprehensively investigated for cancer and pre-cancer diagnosis and evaluation in humans [7–10]. These investigations showed that specific spectral features of Raman spectra could be used to correlate with the molecular and structural changes of tissue associated with neoplastic transformation [6–14]. Near-infrared Raman spectroscopy has also been applied to *in vivo* pre-cancer and cancer diagnosis and detection in organs such as breast, stomach, skin, lung and cervix [10, 12–14]. At the same time, some new methods of chemometrics, such as supported vector machines (SVM) [15, 16], have been developed, by which we can analyze the Raman spectra of biomedical sample more accurately.

In the present research, we used a Fourier transformation near infrared (FT-NIR) Raman spectroscope to detect samples of normal oral mucosa, OSCC and mild, moderate, and severe dysplasia. Subsequently, radial basis function SVM was carried out to classify the Raman spectra of different groups and establish the discriminating model. The efficiency of this algorithm was evaluated by specificity, sensitivity, accuracy, Matthew coefficient correlation and rigidity. At the same time, principal component analysis (PCA) and linear discriminant analysis (LDA) were used to verify the results of SVM. The aim of the present research was to discriminate oral dysplasia of different stages by the near-infrared Raman spectroscope, in order to evaluate the histological classification system of oral dysplasia.

## RESULTS/DISCUSSION

Up to date, the pathological diagnosis has remained to be the “golden standard” in the clinical works. But it is a subjective method based on the pathologists’ experiences, and different pathologists may reach different diagnoses for the same HE section sometimes, especially for grading the dysplasia of the premalignant lesion [3]. So in grading of the dysplasia, we invited three experienced observers to classify the dysplasia blindly, in order to degrade the intra- and inter-examiner variability as possible as we could. The samples consisted of 46 normal, 20 OSCC and 88 dysplastic areas. Of the 88 dysplastic areas, 40 areas were identified as mild dysplasia, 16 as moderate dysplasia, and 32 as severe dysplasia. All the areas were marked in the Raman spectral sections.

FT-NIR Raman spectroscopy shows some advantages in the diagnosis of oral diseases [17]: firstly, we could not completely remove the disturbance from saliva, so it is important that the water absorption in FT-NIR Raman spectroscopy does not disturb the measurement, in contrast to FT-IR spectroscopy; in the second, using excitation at 1064 nm by an Nd:YAG laser virtually eliminates fluorescence; and the usually high signal-to-noise ratio of the FT-NIR Raman spectra makes the chemometric methods applicable; what's more, it is most important for the classification of oral dysplasia that the NIR Raman spectroscopy is objective, which is based on the biochemical variations in the tissue samples. In previous studies [6, 7, 9–11, 13, 14, 18, 19], FT-NIR Raman spectroscope was used to detect and discriminate normal tissues, premalignant lesions, benign and malignant tumors of oral mucosa, skin, stomach mucosa, breast, liver and cervical mucosa etc. Combined with chemometric methods, the efficiency of the strategy was satisfactory, and suggested a great opportunity for using NIR Raman spectroscopy in medical research and clinical applications. So we could use it to evaluate the WHO classification system of oral dysplasia.

**Figure 1:**
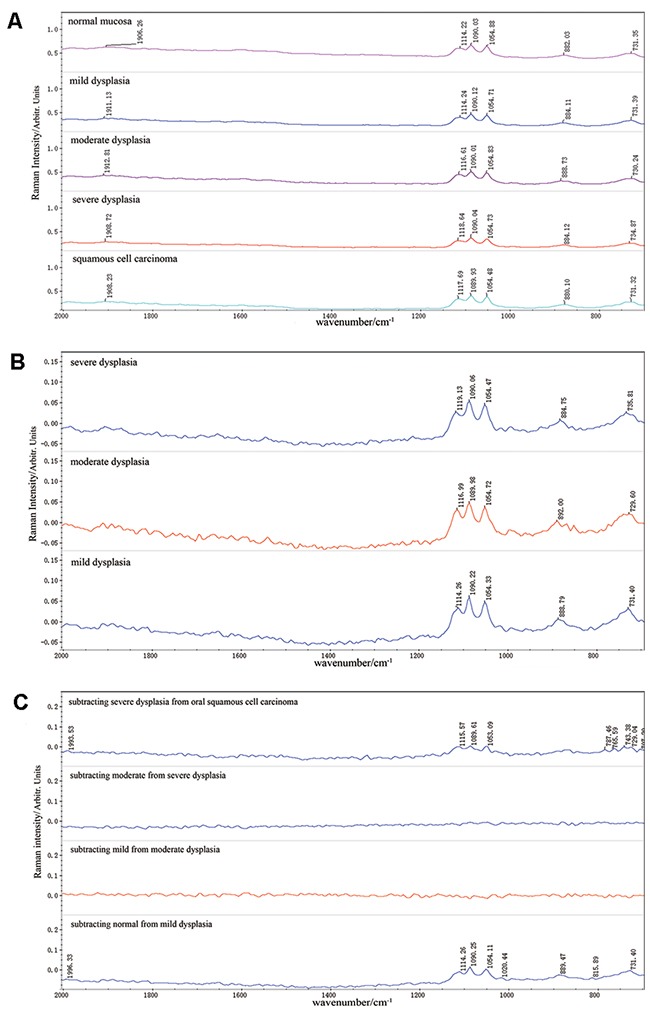
Raman Spectra of different analytic methods **(A)** The mean spectra of normal mucosa, mild, moderate and severe dysplasia, OSCC. **(B)** The variations between the mean spectra of three grades of dysplasia and the normal mucosa. **(C)** Variations by subtracting the mean spectra of lower histopathological grades from the higher ones.

Raman spectra were acquired from all samples successfully. We used the mean spectra of the normal mucosa, OSCC and dysplasia of the three grades, in order to eliminate the variation of a single spectrum and maintain commonality. Using OMNIC 8.0 software, the resultant mean spectra of normal mucosa, OSCC, mild, moderate and severe dysplasia can be seen in Figure 1A. The tentative biochemical assignments of individual peaks, which were automatically indentified by OMNIC 8.0, were given in Table 1 [6, 9, 18, 20, 21]. On the first examination, the spectra were visually similar but detailed examination revealed noticeable variations that could give an insight into the biochemical changes taking place. It was found that the main peaks appeared in the region of 800-1800cm^-1^, the so-called “fingerprint region” [10]. The most significant peaks existed in 730, 884, 1054, 1090 and 1116cm^-1^, and the other peak at 1911 cm^-1^ was not so prominent. In these peaks, the 730, 884, 1054, 1090cm^-1^ were assigned to the DNA, the 1116 and 1911cm^-1^ were assigned to the protein, which were consistent with the epithelial origin of the samples. To confirm that the subtle differences in the mean Raman spectra were in fact variations in the collected Raman signals and not the result of background fluorescence, fluctuations or noise, the variance in the data was calculated according to spectral position.

**Table 1:** Peak assignment in Raman spectra

Normal	Mild	Moderate	Severe	OSCC	Peak assignment
731.35	731.39	730.24	734.87	731.32	Basic group in DNA
882.03	884.11	888.73	884.12	880.10	Deoxyribose phosphate chain vibration
1054.88	1054.71	1054.83	1054.73	1054.48	C-O stretching in DNA
1090.03	1090.12	1090.01	1090.04	1089.93	DNA: O-P-O backbone stretching
1114.22	1114.24	1116.61	1118.64	1117.69	C–N stretching in protein
1906.26	1911.13	1912.81	1908.72	1908.23	ν(C-O) in tyrosine

Figure 1B shows the variations between the mean spectra of three grades of dysplasia and the normal mucosa. It was interesting that all the variations existed in the same wavenumbers of 730, 884, 1054, 1090, 1116cm^-1^, except for some peak shifts and decomposition, which were assigned to the DNA and protein. These variations might demonstrate the high proliferative ability of the dysplastic cells. In order to observe the biochemical changes in the dysplastic progression, we subtracted the mean spectra of lower grades from the higher ones and showed the results in Figure 1C. It was remarkable that the differences between the dysplastic grades were minor and no significant peaks were observed in the background. What's more, there were not any gradual changes correlated to the dysplastic stage. These results demonstrated that there were not significant biochemical variations among the dysplastic stages of WHO classification. Compared with the above results, the variations between mild dysplasia and normal mucosa were relatively significant and sharp peaks in 731.40, 815.89, 888.79, 1020.44, 1054.11, 1090.25, 1114.26 and 1996.33cm^-1^ were observed. It could be concluded that the biochemical variations between oral dysplasia and normal mucosa were larger than those among different dysplastic stages. The similar results could be observed between the severe dysplasia and OSCC.

Many methods have been explored by researchers to analyze and classify the Raman spectra of different tissue samples [10, 22]. Malini R *et al.* [23] applied principal component analysis combined with multiparameter limit tests to allow match/mismatch criteria to be applied to test normal, inflammatory, premalignant, and malignant conditions in oral tissue. Sensitivity and specificity were 100% for the diagnosis of the malignancy. In the present research, SVM were employed for the classification of the spectra, since it offers the capability of learning nonlinear arithmetic operations based on a training set and can generalize a compact model, which can later be applied to unknown spectra of interest [15, 16]. In the previous research [24], we detected the biochemical variations in oral tissues that were normal, premalignant and malignant, and established diagnostic models accurately by FT-NIR Raman spectroscope combined with SVM. But in the pilot study of this research, in which the whole spectra of 100~3800 cm^-1^ were used as inputs, the accuracies of comparison between mild dysplasia and normal, moderate dysplasia and normal, severe dysplasia and normal were 52.5%, 0%, 71.88%, which elucidated the low modeling ability of support vector machines (unpublished data).

The WHO classification system of oral dysplasia is focusing on the risk of progression to malignancy. So in the present study, we used Gaussian radial basis function SVM to discriminate OSCC and oral dyaplasia of different grades. The results showed that this strategy was not so sensitive to group and model the lesions. Using the optimized parameters of gamma=0.002967359 and cost=1, all mild dysplastic spectra were classified correctly (40/40), and so were all the OSCC spectra (20/20). It was surprising that the algorithm could not establish the model of moderate dysplasia. Only two moderate dysplastic spectra were classified correctly (2/16), and fourteen OSCC spectra were classified accurately (14/20). In discriminating the severe dysplasia and OSCC group, the accuracy of this algorithm was not good. Ten OSCC spectra were classified to the OSCC set (10/20), and twenty-seven severe dysplastic spectra were classified correctly (27/32). Based on the above results, the specificity, sensitivity, accuracy, Matthew coefficient correlation and rigidity were calculated and are shown in Table 2.

**Table 2:** Classification parameter of mild, moderate, severe dysplasia and OSCC

Parameters	Mild and OSCC^a^	Moderate and OSCC^a^	Severe and OSCC^a^
Sp	100%	70%	50%
Se	100%	12.5%	84.38%
Acc	100%	44.44%	71.15%
MCC	1	-0.21	0.37
error	0	0.3	0.5
R	1	0.25	0.51

Note: ^a^ spectra of dysplasia as the positive sample.

**Figure 2:**
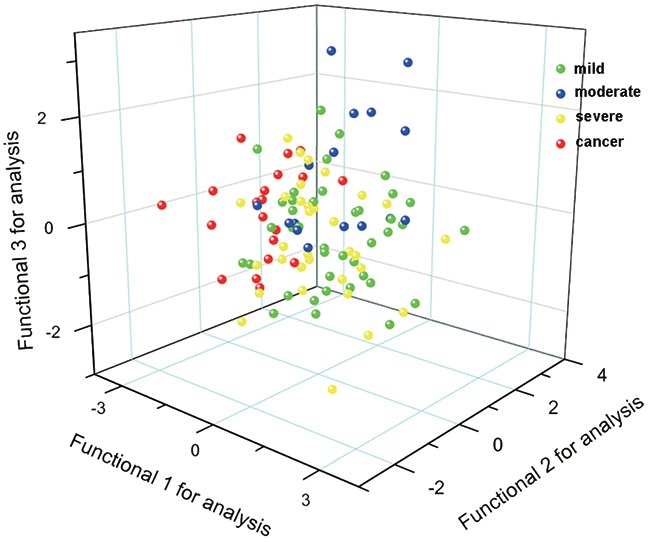
3-D scatter plots of PCA-LDA analysis

The above results of SVM were interesting, especial for the moderate dysplasia. In order to further evaluate the accuracy of WHO histological classification, we used SVM to discriminate the three grades inside the dysplastic group. When discriminating the mild dysplasia and other groups containing moderate and severe dysplasia, thirty-seven mild spectra were classified correctly (37/40) and so were forty-six spectra of other groups (46/48). Twenty-six severe spectra (26/32) and forty-five other ones containing mild and moderate dysplasia (45/56) were discriminated correctly. Based on the above results, the specificity, sensitivity, accuracy, Matthew coefficient correlation and rigidity were calculated and are shown in Table 3. It could be concluded that the moderate dysplasia might bring confusion to the grading system, and there might be some biochemical variations between the mild and moderate-severe dysplasia. In the 3-D scatter plots of PCA-LDA analysis (Figure 2), we could not observe any significant boundaries among the plots of different dysplastic grades. According to the WHO histological classification, the plots of moderate dysplasia should locate between those of the mild and severe ones, but five ones were far from the center of the plots. This results was similar to that of SVM, so we suggested that the moderate dysplasia was not a middle stage between the mild and severe, and there were not significant biochemical variations in the three dysplastic grades.

**Table 3:** Classification parameter of the calibration set inside oral dysplasias

Parameters	Mild and others^a^	Severe and others^b^
Sp	95.83%	80.36%
Se	92.5%	81.25%
Acc	94.32%	80.68%
MCC	0.89	0.60
error	0.08	0.20
R	0.93	0.76

Note: ^a^ spectra of mild dysplasia as the positive sample; ^b^ spectra of severe dysplasia as the positive sample.

A histological dysplasia system ideally should be easily applicable in daily routine practice with low inter and intra-observer variability. Several studies have shown great inter- and intra-observer variability in the grading of oral dysplasia by the WHO classification, with the kappa scores of 0.125 to 0.59 [4, 25–28]. Considering the problems in making reliable distinctions between the different grades, the Working Group of WHO considered collapsing the four grades to two when reporting the presence or absence of epithelial dysplasia: “no/questionable/mild”-low risk, ”moderate or severe”-implying high risk. The utility of this was recently tested and has been shown to have merit in that better agreement was reached between those experienced in examining oral biopsies with improvement in kappa agreements [29]. But the binary grading system was still based on the architectural and cytological changes, which relied on the observation of pathologists and was subjective. Future discoveries mainly in molecular biology, such as Raman spectroscopy, could be the basis for a single, universal classification system for intraepithelial lesions for the oral cavity.

The present research was carried out to evaluate the WHO classification system by the NIR Raman spectroscope, which is an objective method to detect the biochemical variations. And the results showed that there were no significant or gradual variations between the different dysplastic grades. Especially for the moderate grades, the diagnostic model cannot be established by SVM. So we deemed that the WHO grading system needed further improvement based on the biochemical analysis, but not only on the pathological manifestation.

## MATERIALS AND METHODS

### Ethics statement

This study has been approved by the Institutional Review Board of West China School of Stomatology. Informed consent was obtained from the subjects. The Declaration of Helsinki protocols were followed during the whole study.

### Patients

From 2007 to 2009, a total of forty patients with OLK and ten patients with OSCC were randomly selected to participate in the present research. The normal samples of twenty-three patients were obtained from the surgical margin in the tumor surgery, or from the excess mucosa in the trauma or orthognathic surgery. A thorough review of clinical records was performed. Patient ages, gender, primary sites of the lesion were recorded. The demographics of the patients are shown in Table 4. None of the OLK and OSCC patients had received any treatments for 3 months prior to the research, had uncontrolled infection, or had immuno-deficiency disease. All the pathological diagnoses were carried out with hematoxylin-eosin (HE) slides by experienced pathologists according to the 2005 WHO histological classification [2].

**Table 4:** Numbers of pathological sections and Raman scanning points

Case information		Normal	Severe	Moderate	Mild	OSCC
All patients		23	14	10	16	10
Age		Range28~54 yrs	Range32~56 yrs	Range30~55 yrs	Range 31~54 yrs	Range29~55 yrs
		Median 41yrs	Median 41.5yrs	Median 40yrs	Median 43yrs	Median 42yrs
Gender	Male	11	6	4	7	5
	Female	12	8	6	9	5
Primary site	Tongue	10	7	6	8	6
	Bucca	13	7	4	8	4
Raman spectra		46	32	16	40	20

### Tissue samples

The normal tissue samples were fixed by 10% formalin and embedded in paraffin. Formalin-fixed paraffin preserved (FFPP) tissue samples of OLK and OSCC were obtained from the Department of Pathology, West China Hospital of Stomatology, Sichuan University. Five parallel 5-μm FFPP sections were cut from each block using a microtome, and one of them was selected randomly to be mounted on glass slides and dried. Samples were dewaxed in-house prior to investigation by immersion in baths of Xylene (BDH), Ethanol Absolut (Merck) and Industrial Methylated Spirits 95% (Lennox) and air-dried. The reference section from each sample was stained with hematoxylin and eosin. Another five parallel 10-μm FFPP sections were cut from each block using a microtome, and one of them was selected randomly to mount on custom CaF_2_ chips, dewaxed and air-dried (Raman spectral sections). These sections were kept unstained for spectroscopic examination. All the tissue sections were characterized by three experienced pathologists blindly, according to the 2005 WHO classification [2]. The grades of dysplasia were determined only if all the three pathologists reached the same results, otherwise the pathological regions were abandoned.

### Instrument

We used a Nicolet Nexus 670 NIR Raman spectroscope (Thermo Nicolet Co., USA) to detect Raman spectra of tissue samples. Radiation of 1064 nm and 1000 mW from an Nd: YAG laser was used for excitation. Sections were placed in the sample window and the laser beam was focused on the biopsy in a spot *ca.* 100 um in diameter. For each biopsy 256 scans were accumulated with a resolution of 8 cm^-1^. OMNIC 8.0 software (Thermo Fisher Scientific Inc., USA) was used to perform baseline correction, obtain mean spectra and subtract the mean spectra of different groups.

### Data analysis

Analysis of the spectra was carried out by two methods. Initially, spectra of normal, OSCC and dysplastic samples were visually inspected. The subtracted mean Raman spectra of difference groups were observed, and the peaks of wave numbers in the spectra were assigned to different biochemical variation, based on previous researches [6, 9, 18, 20, 21].

In the second step, the original SPA format was transferred to CSV format, and all the spectra were smoothed by wavelet transform, using bior 4.4 of the Matlab program, and the number of resolution layers was two. The LIBSVM software (http://www.csie.ntu.edu.tw/~cjlin/libsvm/) was used to carry out the radial basis function SVM, with the optimized parameters of gamma=0.002967359 and cost=1. The spectra of the paired groups of OSCC and mild dysplasia, OSCC and moderate dysplasia, OSCC and severe dysplasia were used to test the discriminating efficiency of SVM. At the same time, we established another two paired groups to test SVM, including the mild dysplasia with moderate and severe ones, and the severe with the mild and moderate ones. The SVM model was presented with the spectra of the regions from 700 to 2000cm^-1^ without principal component analysis for preprocessing. The performance of the model was evaluated during training by cross-validation, in which some of the spectra were left out and used for testing how well the model could predict unseen spectra. Owing to the limited number of spectra in this study, only one spectrum of different groups was left out. This process of ‘leaving one out’ was repeated so that each spectrum was left out for cross-validation exactly once. The Raman intensities at the 338 different wavenumbers equally spread between 700 and 2000 cm^-1^ were used as inputs.

Five parameters were chosen to test the efficiency of the algorithm: Specificity (Sp), Sensitivity (Se), Accuracy (Acc), Matthew correlation coefficient (MCC) [30] and rigidity (R)[31]. Their formula were as follow (Note: TP: true positive; FP: false positive; TN: true negative; FN: false negative):

Specificity: $Sp=\frac{TN}{TN+FP}$

Sensitivity: $Se=\frac{TP}{TP+FN}$

Accuracy: $Acc=\frac{TP+TN}{TP+TN+FP+FN}$

Matthew correlation coefficient: $MCC=\frac{TP\times TN-FP\times FN}{\sqrt{\left(TP+FN)\times (TP+FP)\times (TN+FP)\times (TN+FN\right)}}$

Rigidity: $error=\frac{FP}{FP+TN};\;R=\frac{2\times (Acc-error)}{1+|Acc-error|}$

To testify the results of SVM for differentiating the OSCC and dysplastic samples, principal component analysis (PCA) combined with linear discrimination analysis (LDA) was employed on the oral mucosa Raman spectra through SPSS 13.0 software package (SPSS Inc, Chicago). PCA is a commonly used data reduction technique in statistics, which can simplify the complex data and extract the key variables as the principal components (PCs). In the process of PCA, a total of 16 PCs was extracted from the Raman spectral data and first three PCs account the largest variance (86%). All the 16 PCs account cumulative 91% of the variance. Then according to PC scores, LDA was used to develop diagnostic algorithm to classify the different spectra. In the process of LDA, leave-one-out and cross-validation method were performed, and three canonical discriminant functions were used. Figure 2 shows the 3-D scatter plot with three canonical discriminant functions as the three axes.

## Abbreviations

OSCC: oral squamous cell carcinoma
OLK: oral leukoplakia
SVM: support vector machines
PCA: principal component analysis
LDA: linear discriminant analysis
DNA: deoxyribose nucleic acid
FT-NIR: Fourier transformation near infrared
HE: hematoxylin and eosin
FFPP: formalin-fixed paraffin preserved
WHO: World Health Organization
Sp: specificity
Se: sensitivity
Acc: accuracy
MCC: Matthew correlation coefficient
R: rigidity

## References

[1] Pindborg JJ, Reichart PA, Smith CJ, van der Wall I (1997). World Health Organization: Histological Typing of Cancer and Precancer of the Oral Mucosa.

[2] Gale N, Plich BZ, Sidransky D, Westra W, Califano J, Barnes L, Eveson JW, Reichart P, Sidransky D (2005). Tumours of the hypopharynx, larynx and trachea (Epithelial precursor lesions). World Health Organization Classification of Tumours Pathology & Genetics Head and Neck Tumours International Agency for Research on Cancer (IARC).

[3] Warnakulasuriya S, Reibel J, Bouquot J, Dabelsteen E (2008). Oral epithelial dysplasia classification systems: predictive value, utility, weaknesses and scope for improvement. J Oral Pathol Med.

[4] Fleskens S, Slootweg P (2009). Grading systems in head and neck dysplasia: their prognostic value, weaknesses and utility. Head Neck Oncol.

[5] Bosman FT (2001). Dysplasia classification: pathology in disgrace?. J Pathol.

[6] Rehman S, Movasaghi Z, Tucker AT, Joel SP, Darr JA, Ruban AV, Rehman IU (2007). Raman spectroscopic analysis of breast cancer tissues: identifying differences between normal, invasive ductal carcinoma and ductal carcinoma in situ of the breast tissue. J Raman Spectrosc.

[7] Stone N, Hart Prieto MC, Crow P, Uff J, Ritchie AW (2007). The use of Raman spectroscopy to provide an estimation of the gross biochemistry associated with urological pathologies. Anal Bioanal Chem.

[8] Tang HW, Yang XB, Kirkham J, Smith DA (2007). Probing intrinsic and extrinsic components in single osteosarcoma cells by near-infrared surface-enhanced Raman scattering. Anal Chem.

[9] Kast RE, Serhatkulu GK, Cao A, Pandya AK, Dai H, Thakur JS, Naik VM, Naik R, Klein MD, Auner GW, Rabah R (2008). Raman spectroscopy can differentiate malignant tumors from normal breast tissue and detect early neoplastic changes in a mouse model. Biopolymers.

[10] Teh SK, Zheng W, Ho KY, Teh M, Yeoh KG, Huang Z (2008). Diagnostic potential of near-infrared Raman spectroscopy in the stomach: differentiating dysplasia from normal tissue. Br J Cancer.

[11] Mahadevan-Jansen A, Mitchell MF, Ramanujam N, Malpica A, Thomsen S, Utzinger U, Richards-Kortum R (1998). Near-infrared Raman spectroscopy for in vitro detection of cervical precancers. Photochem Photobiol.

[12] Pappas D, Smith BW, Winefordner JD (2000). Raman spectroscopy in bioanalysis. Talanta.

[13] Caspers PJ, Lucassen GW, Puppels GJ (2003). Combined in vivo confocal Raman spectroscopy and confocal microscopy of human skin. Biophys J.

[14] Huang Z, McWilliams A, Lui H, McLean DI, Lam S, Zeng H (2003). Near-infrared Raman spectroscopy for optical diagnosis of lung cancer. Int J Cancer.

[15] Vapnik VN (1995). The Nature of Statistical Learning Theory.

[16] Webb AR (2004). Statistical Pattern Recognition.

[17] Schrader B, Fendel S, Keller S, Lochte T, Riedl M, Schulte R, Tatsch E, Dippel B (1997). NIR FT Raman spectroscopy- a new tool in medical diagnostics. J Mol Struct.

[18] Jess PR, Smith DD, Mazilu M, Dholakia K, Riches AC, Herrington CS (2007). Early detection of cervical neoplasia by Raman spectroscopy. Int J Cancer.

[19] Lyng FM, Faolain EO, Conroy J, Meade AD, Knief P, Duffy B, Hunter MB, Byrne JM, Kelehan P, Byrne HJ (2007). Vibrational spectroscopy for cervical cancer pathology, from biochemical analysis to diagnostic tool. Exp Mol Pathol.

[20] Short KW, Carpenter S, Freyer JP, Mourant JR (2005). Raman spectroscopy detects biochemical changes due to proliferation in mammalian cell cultures. Biophys J.

[21] Andrade PO, Bitar RA, Yassoyama K, Martinho H, Santo AM, Bruno PM, Martin AA (2007). Study of normal colorectal tissue by FT-Raman spectroscopy. Anal Bioanal Chem.

[22] Li Y, Wen ZN, Li LJ, Li ML, Gao N, Guo YZ (2010). Research on the Raman spectral character and diagnostic value of squamous cell carcinoma of oral mucosa. J Raman Spectrosc.

[23] Malini R, Venkatakrishna K, Kurien J, Pai KM, Rao L, Kartha VB, Krishna CM (2006). Discrimination of normal, inflammatory, premalignant, and malignant oral tissue: a Raman spectroscopy study. Biopolymers.

[24] Lieber CA, Majumder SK, Billheimer D, Ellis DL, Mahadevan-Jansen A (2008). Raman microspectroscopy for skin cancer detection in vitro. J Biomed Opt.

[25] Abbey LM, Kaugars GE, Gunsolley JC, Burns JC, PageDG Svirsky JA, Eisenberg E, Krutchkoff DJ, Cushing M (1995). Intraexaminer and interexaminer reliability in the diagnosis of oral epithelial dysplasia. Oral Surg Oral Med Oral Pathol Oral Radiol Endod.

[26] Karabulut A, Reibel J, Therkildsen MH, Praetorius F, Nielsen HW, Dabelsteen E (1995). Observer variability in the histologic assessment of oral premalignant lesions. J Oral Pathol Med.

[27] Tabor MP, Braakhuis BJ, van der Wal JE, van Diest PJ, Leemans CR, Brakenhoff RH, Kummer JA (2003). Comparative molecular and histological grading of epithelial dysplasia of the oral cavity and the oropharynx. J Pathol.

[28] Fischer DJ, Epstein JB, Morton TH, Schwartz SM (2004). Interobserver reliability in the histopathologic diagnosis of oral pre-malignant and malignant lesions. J Oral Pathol Med.

[29] Kujan O, Oliver RJ, Khattab A, Roberts SA, Thakker N, Sloan P (2006). Evaluation of a new binary system of grading oral epithelial dysplasia for prediction of malignant transformation. Oral Oncol.

[30] Matthews BW (1975). Comparison of the predicted and observed secondary structure of T4 phage lysozyme. Biochim Biophys Acta.

[31] Novic M, Zupan J (1995). Investigation of infrared spectra-structure correlation using Kohonen and counterpropagation neural network. J Chem Inf Comput Sci.

